# The rise and fall of Queckenstedt's test between 1916 and 1970, a milestone in spinal cord diagnostics and why it matters

**DOI:** 10.1111/ene.16556

**Published:** 2024-11-27

**Authors:** Armin Curt, Carl Moritz Zipser

**Affiliations:** ^1^ Spinal Cord Injury Center Balgrist University Hospital Zurich Switzerland

**Keywords:** cerebrospinal fluid pressure, compressive myelopathy, spinal cord compression, spinal cord injuries

## Abstract

**Background and purpose:**

In 1916, Hans H. G. Queckenstedt (1876–1918) was the first to describe a test aimed at detecting spinal cerebrospinal fluid (CSF) space obstruction through lumbar CSF pressure measurements in paraplegic patients. For this test, bilateral jugular vein compression was applied during lumbar puncture and consecutive changes in lumbar CSF pressure were then observed. Findings were rated as normal, or indicative of incomplete or complete spinal block. This test, known as Queckenstedt's test, became widely adopted and further developed in the field.

**Methods:**

This systematic literature review provides an overview of the milestones in research using Queckenstedt's test.

**Results:**

Clinical research involving Queckenstedt's test was widely disseminated across the globe. In 1922, the proof of concept for Queckenstedt's test was provided by James B. Ayer (1882–1963) through simultaneous cisternal and lumbar CSF pressure measurements. He found that the cisternal (in contrast to lumbar) pressure remained responsive in cases with spinal block. The test was further refined up until the 1960s, and was considered a routine diagnostic procedure for testing of spinal canal obstruction. Developments in non‐invasive spinal computed tomography and magnetic resonance imaging led to a significant decline in interest in Queckenstedt's test, and the test eventually disappeared from textbooks and clinical routine. However, at the beginning of the 21st century there was renewed interest in revealing the biomechanical properties of the CSF through advanced recording and computational techniques to complement spinal imaging.

**Conclusion:**

Spine and spinal cord physicians should be familiar with Queckenstedt's test, which not only represented a milestone in spinal diagnostics, but provided a physiological framework for the appreciation of spinal cord compression that is still valid today.

## INTRODUCTION

In 1916, Hans H. G. Queckenstedt (1876–1918), a neurologist from Rostock, Germany, published a seminal paper on a bedside test for detecting cerebrospinal fluid (CSF) space obstruction [1]. Soon after, the test was named after him and attracted international attention. Most resources, such as Merriam‐Webster's Medical Dictionary [2] and Adams and Victor's Principles of Neurology [3], refer to it as Queckenstedt's test, and it is sometimes called Queckenstedt's sign or maneuver. The Medical Literature Analysis and Retrieval System, as established by the National Library of Medicine, described it as follows:

“Queckenstedt‐Stookey maneuver”.

“Syn. Queckenstedt sign”.

“When the veins in the neck are compressed on one or both sides there is a rapid rise in the pressure of the cerebrospinal fluid of healthy persons, and this quickly disappears when pressure is taken off the neck. But when there is a block in the vertebral canal the pressure of the cerebrospinal fluid is little or not at all affected (Dorland)”.

Recent historical vignettes of Hans Queckenstedt focus on his career [4]. However, the history of Queckenstedt's test is untold, from the adventurous decades when it was eventually employed as a routine test in spinal neurology, until its disappearance after advances in non‐invasive spinal cord imaging. The aim of this systematic review was to summarize the milestones in Queckenstedt's test research, and to trace the global dissemination and reception of this test. Ultimately, this review will introduce the reader to the concepts behind the different synonyms for the “Queckenstedt‐Ayer‐Stookey‐Tobey” test.

## METHODS

This systematic historical review was conducted according to the “PICO” method: population (P): patients with spinal cord diseases; intervention (I): Queckenstedt's test; comparison (C): operative findings, post‐mortem analyses, protein count, and myelography; and outcome (O): Queckenstedt's test findings. The review was registered in PROSPERO (CRD42024498900). Research questions were milestones of Queckenstedt's test research and dissemination. The search strategy was: ((queckenstedt) OR (queckenstedt's test)) OR (queckenstedt's manoeuvre). Further studies were identified through cross‐referencing. All types of original work, textbook chapters, or monographs were considered. Case reports were included to answer the research question regarding dissemination of Queckenstedt's test. Accounting for the variety of scientific languages in the beginning of the 20th century, full texts in English, French, German, and Italian were considered. The search was performed on August 15, 2024, in MEDLINE and PubMed, as well as in Swisscovery, a national platform that brings together scientific information from approximately 500 libraries in Switzerland.

## RESULTS

### Search results

In total, there were 228 search results from MEDLINE (*n* = 23), PubMed (*n* = 73) and Swisscovery (*n* = 132). After removal of duplicates and articles not associated with Queckenstedt's test for spinal cord diseases, 22 relevant milestone studies were selected for this review, 10 of which were selected to create a top reading list. Cohort studies were mostly published from European and North American centers. From European centers, there were research articles originating from Canada, Denmark, France, Germany, Italy, Japan, Netherlands, Norway, and Sweden, with further case reports originating from France, Italy, Japan, Poland, and Russia (in alphabetical order). Journals included those from the top tiers in neurology and neuroscience, such as *Archives of Neurology and Psychiatry* (the predecessor of *JAMA Neurology*), *JAMA*, *The Lancet*, *Brain*, and *Deutsche Zeitschrift für Nervenheilkunde* (the predecessor of *Journal of Neurology*). Most studies were published between the 1920s and 1960s.

### Initial description of Queckenstedt's test and its early dissemination

**FIGURE 1:**
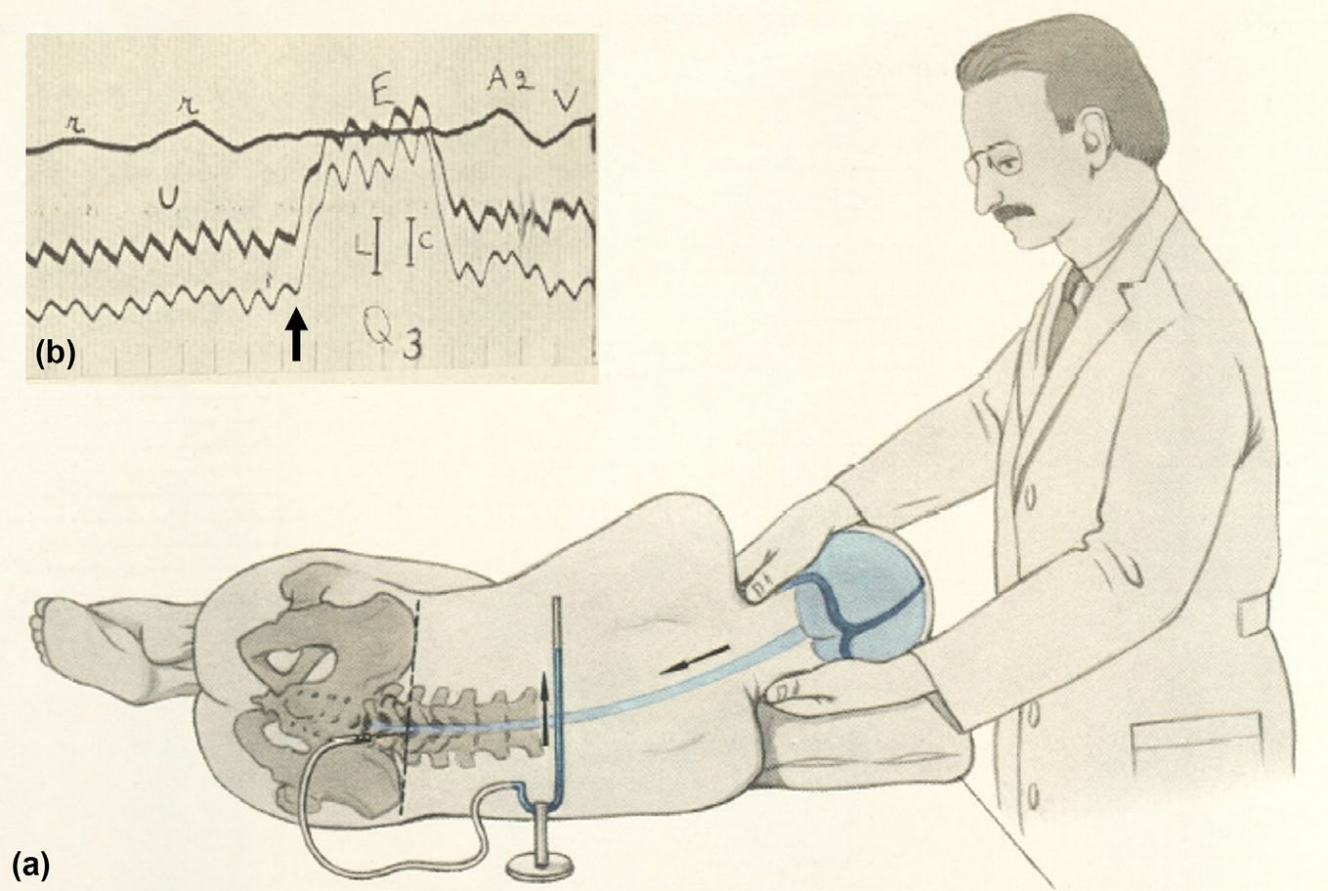
(a) Queckenstedt's test performed by Hans Queckenstedt with manometric response. (b) Early optical registration of Queckenstedt's test by Sten Lagergren (Q3; arrow added) at cisternal level (middle curve) and lumbar level (bottom), with parallel measurement of respiration (top curve). Patient without cerebrospinal fluid space constriction.

**FIGURE 2:**
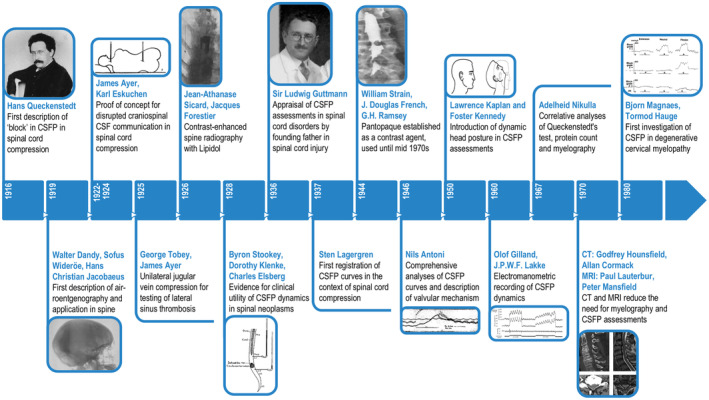
Milestones in the diagnosis of spinal cord compression. CSF, cerebrospinal fluid; CSFP, cerebrospinal fluid pressure; CT, computed tomography; MRI, magnetic resonance imaging.

The recognition that jugular vein compression was associated with CSF pressure increase was described before Queckenstedt's time in post‐mortem studies in 1863 (cited after Sten Gustaffson Lagergren) [5] and in human studies in 1901 [6], but Queckenstedt is recognized for being the first to translate this test to spinal cord compression (Figure 1). Before Queckenstedt's test, there was a research focus on the chemical properties of CSF, namely, protein elevation, for diagnosing spinal cord compression in para‐ and tetraplegia [7]. However, a significant number of patients had protein elevations for reasons other than spinal cord compression (e.g., multiple sclerosis). In 1916, Queckenstedt highlighted the diagnostic value of the impaired biomechanical properties of the CSF initially described in five patients with para‐ and tetraplegia, and this discovery was then rapidly disseminated across Europe and North America (Figure 2).

The dissemination of this discovery in the United States was owed to James B. Ayer (1882–1963). He contributed significantly to the development of cisternal puncture [8] and combined it with parallel lumbar pressure measurements in patients with suspected spinal cord tumor in 1922 [9]. As a proof of concept of Queckenstedt's test, he demonstrated, through parallel measurements in 65 patients with spinal cord tumor, that cisternal CSF pressure would rise, whereas lumbar CSF pressure would not. This discovery earned him his portion of the eponym “Queckenstedt–Ayer” test. Notably, independently of Ayer, Karl Eskuchen (1885–1955) established cisternal puncture in Munich and Zwickau, Germany and, in 1924, published parallel cisternal and lumbar CSF pressure assessments in patients with suspected spinal canal obstruction [10]. Ayer established a CSF laboratory at Massachusetts General Hospital, conducted over 2000 cisternal punctures, and continued research [11]. There, together with George L. Tobey (1881–1967), he used Queckenstedt's test for the diagnosis of lateral sinus thrombosis. In their initial description of seven cases, they applied bilateral or unilateral jugular vein compression during lumbar puncture [12]. In 1925, it was found that unilateral compression on the affected side would not lead to as high a rise in CSF pressure on the healthy side; this became the “Tobey–Ayer” test.

### Advanced clinical research and routine application

In the mid‐ to late 1920s, substantial evidence for clinical utility was mainly provided by the US neurosurgeon Byron Stookey (1887–1966) and Charles Albert Elsberg (1871–1948), who was one of the Chiefs of Service of the Neurological Institute in New York and a pioneer in the field of spinal cord injury [13]. In his first study on manometric tests, Stookey reported data from 50 patients with suspected spinal tumor collected between 1921 and 1925 [14]. In this study, he also described a modified Queckenstedt's test, carried out with bilateral slight compression of the jugular veins, so‐called “touch compression”, as compared to deep compression, to reduce confounding through coughing and movement [15]. This was followed by another milestone study by Stookey and Dorothy Klenke Nash (1898–1976) in 235 patients with suspected spinal tumor [16, 17]. These authors were the first to provide sizable cohort data to determine diagnostic accuracy. Of 85 patients who had a positive Queckenstedt's test, 45 were operated on, and all had tumors, most commonly extra‐ (*n* = 14) or intradural tumors (*n* = 18). Of 125 patients who had a negative Queckenstedt's test, 10 patients were referred to surgery. None of these patients had operative findings pointing towards spinal canal obstruction. In doubtful cases, the authors advocated performing simultaneous cisternal and lumbar puncture. The Queckenstedt–Stookey test is probably the most common eponymous test for manometric assessment of spinal cord compression, even when unrelated to touch compression. This can be explained, first, by the high relevance of Stookey for broader clinical application. This could be why clinicians and researchers interested in spinal disorders selected the Queckenstedt–Stookey eponym over Ayer or Tobey, despite the equal importance of their work. Second, the evolution of medical eponyms is also subject to cultural and language factors, which warrant further investigation in this case [18, 19]. Elsberg published findings from another 119 operations in spinal cord tumor stating that “manometric studies of the spinal fluid… have contributed greatly to improvement in our diagnosis…” [15]. Generally, Queckenstedt's test results were reported in studies as “normal”, “complete (spinal) block” or “incomplete/partial/relative (spinal) block”. A normal Queckenstedt's test was characterized by a rapid rise and fall of CSF pressure, whereas in a complete block, CSF pressure response would be absent. Incomplete block was characterized by a slow rise or valvular effect with persisting increased or only slowly decreasing CSF pressure after release of jugular compression, as initially described by Nils Antoni (1887–1968) from the Karolinska Institute, Stockholm [20]. His work represents one of the earliest comprehensive and thorough analyses of CSF pressure curves in relation to spinal cord compression and physiological features (e.g., respiration). Antoni investigated abnormal respiratory modulation of CSF pressure as an indicator of spinal block (“Antoni breathing probe”); he considered impaired venous translation of respiratory modulation as the underlying physiological concept, cited after one of his scholars, Sten Gustaffson Lagergren (1900–1973) [5]. Lagergren should be recognized for one of the most comprehensive monographs on the early history and physiology of Queckenstedt's test and for being one of the first to facilitate optical registration of Queckenstedt's test, which had previously been carried out with manometers (Figure 1b). In the following decades, Queckenstedt's test was mostly applied when tumor was suspected. Sir Ludwig Guttmann (1899–1980), a protagonist in spinal cord injury, acknowledged Queckenstedt's test as being important in the diagnosis of spinal cord tumor in a classic neurological textbook [21].

### Queckenstedt's test in the context of novel imaging techniques to diagnose spinal cord compression

In her 1967 dissertation, Adelheid Nikulla, from Cologne, Germany [22], analyzed data from 404 patients. Patients were treated for spinal cord tumor in centers across Germany between 1932 and 1962 (Würzburg, Berlin, Bochum and Cologne). Queckenstedt's test was performed in 335 patients, and protein count and myelography were available in 222 patients. Of these patients, 60% had complete and 26% had partial spinal block. All cases with a pathological Queckenstedt's test had pathological myelography findings. In the 14% of patients with a normal Queckenstedt's test, a spinal cord tumor was diagnosed through myelography. These were angioma at the thoracolumbar level and some glioma. These findings align to a great extent with those of Sassaroli from Rome, Italy (200 patients) [23], Lakke from Groningen, the Netherlands (59 patients) [24], and Gilland, from Sweden (115 patients) [25]. Notably, Gilland and Lakke should be recognized for their historical overview of Queckenstedt's test up to the 1960s. Overall, Queckenstedt's test had high positive predictive value for tumor detection in myelography and higher sensitivity than protein count. The highest sensitivity was found for cervicothoracic intradural extramedullary tumor and metastases. Advances in myelography paralleled research in Queckenstedt's test, with the first description of air‐roentgenography in 1919, and contrast‐enhanced radiography with Lipidol in the 1920s and Pantopaque in the 1940s [26].

### Refinement of Queckenstedt's test

Queckenstedt's test was further refined in the 1940s to 1960s by Kaplan and Kennedy [27] through application of dynamic head positioning to increase sensitivity for incomplete spinal block, through gradual and quantifiable jugular vein compression by Grant and Cone, aiming to improve inter‐trial reliability [28], and through combined abdominal and jugular compression for a higher detection rate of lower thoracic and lumbar tumor [29]. These developments were happening in parallel with further technical advances in continuous visualization of CSF pressure curves [20, 24]. In 1969, a comprehensive monograph by J. P. W. F. Lakke was published, in which he discussed electro‐manometric recordings of the CSF pressure curve and pulsations in over 100 patients with spinal cord compression. Interestingly, studies on Queckenstedt's test from the 1960s to 1970s focused on degenerative cervical spinal canal stenosis [30, 31], where determining the clinical significance of a cervical spinal canal stenosis was identified as a challenge [32].

### From Queckenstedt's test to spine imaging: A paradigm shift

From a history of science point of view, the history of Queckenstedt's test is an excellent example of a paradigm shift according to Thomas S. Kuhn [33]. Fundamentally, there was a transition from manometric, or biomechanical CSF pressure‐derived diagnosis of spinal cord compression to visualization on computed tomography (CT) or magnetic resonance imaging (MRI). Queckenstedt's test initially superseded CSF protein analysis for spinal compression, as it had higher diagnostic accuracy. In the early phase of Queckenstedt research, researchers such as Ayer and Eskuchen provided solidifying evidence, while Stookey provided evidence for clinical utility. Refinements of Queckenstedt's test were made in an attempt to overcome problems such as sensitivity (e.g., increasing sensitivity through head reclination) and reproducibility (e.g., quantifiable jugular vein compression). However, several problems associated with Queckenstedt's test stimulated further research; these were mainly its invasiveness and limited sensitivity, but also the lack of localization information. Myelography did not fully replace Queckenstedt's test, as it was equally invasive, and earlier contrast agents had an unfavorable safety profile [34]. With the advent of improved contrast agents and non‐invasive imaging to visualize the spinal cord, many problems associated with Queckenstedt's test were resolved. The approach towards spinal cord compression underwent a transformation from detecting CSF pressure disturbances to structural correlates of spinal cord encroachment and tumor. The accompanying physiological framework of biomechanical interruption between supra‐ and infra‐stenotic CSF compartments lost relevance in the subsequent period. The shift in paradigm is clearly illustrated by textbook entries over the years. The US neurologist H. Hoston Merritt (1902–1979), a successor of Elsberg at the Neurological Institute of New York, published his monograph “The Cerebrospinal Fluid” in 1937 (with a foreword by James Ayer) [35] and later founded “A Textbook Of Neurology”, from which excerpts of the 4th (1967) [36] and 7th edition (1984) [37] are quoted in the following:

**1937**: “The cerebrospinal fluid findings in cases with a tumor of the spinal cord are essentially those of a complete or partial subarachnoid block. If complete or partial subarachnoid block cannot be demonstrated by lumbar and cistern puncture, the diagnosis of a spinal tumor is only rarely justified, except in cases with symptoms indicating a tumor in the cauda equina.” (p.172).

**1967**: “The diagnosis of an intraspinal tumor can be established […] with the help of certain aids. These include roentgenograms of the spine, spinal puncture with careful testing of the dynamics, and visualization of the subarachnoid space with pantopaque or air.” (p.308)

**1984**: “Before the introduction of myelography, manometry was used to evaluate the patency of the subarachnoid fluid space; a sphygmomanometer cuff placed around the neck and inflated to compress cervical veins. Normally, this would increase CSF pressure, but not if the subarachnoid spaces were compressed by tumor. However, the technique was cumbersome, unreliable, and sometimes dangerous. It is no longer used.” (p.268)

Importantly, Queckenstedt's test does not represent an additional risk to lumbar puncture in general, as there are no known adverse events related to its use. The “danger” referred to in the Merritt textbook most likely relates to Queckenstedt's test performed in patients with elevated intracranial pressure, where lumbar puncture is generally contraindicated [38] and to false‐negative findings that led to misdiagnosis.

## DISCUSSION

To our knowledge, this systematic review represents the first endeavor to trace the evolution of Queckenstedt's test. Queckenstedt's test was a routine diagnostic procedure for diagnosis of CSF space constriction, especially when spinal cord tumor was suspected. Clinical studies commonly aimed to investigate the diagnostic accuracy of Queckenstedt's test as confirmed by operative findings, post‐mortem analyses, myelography, and protein count. Its widespread use confirmed the sensitivity of Queckenstedt's test for revealing spinal block. A seamless comparison to myelography cannot be made, as Queckenstedt's test is aimed at detecting CSF flow disturbances resulting from tumor, whereas myelography facilitates the visualization of tumor. Still, to our best knowledge, findings from myelography and Queckenstedt's test overlap to a large extent. Most failures of Queckenstedt's test were related to tumor at the cranio‐cervical junction and the lumbosacral cord, due to the larger spinal canal size there, as well as small tumors that were not affecting CSF flow [39, 40, 41]. Several studies advanced the value of Queckenstedt's test through refinement and advanced recording methods. We propose a top 10 reading list of the historical literature on Queckenstedt's test [1, 5, 9, 10, 12, 16, 20, 22, 24, 27].

Queckenstedt's test was widely disseminated, with most of the literature originating from Europe and the United States. Many pioneering neurologists and neurosurgeons in the field of spinal cord injury from the first half of the 20th century used Queckenstedt's test in their research. Queckenstedt's test and invasive spinal imaging research were conducted in parallel for several decades. Eventually, Queckenstedt's test for spinal diagnostics was abandoned in favor of non‐invasive imaging techniques (i.e., spinal CT and MRI), providing increasingly detailed structural information of the spine (vertebras), spinal alignment and spinal cord (including epi‐ subdural CSF space obstruction). This said, the history of Queckenstedt's test can also be read as an example of a paradigm shift and a history of modern neurology, from invasive to non‐invasive diagnostic procedures.

### Why does Queckenstedt's test matter today?

The diagnosis of acute and chronic spinal cord injuries nowadays relies on clinical findings and non‐invasive neuroimaging, but the framework behind Queckenstedt's test may still be of interest in the context of degenerative spinal canal stenosis of unclear significance. Novel dynamic imaging techniques were developed to overcome the limitations of anatomical MRI. Among these, non‐invasive phase‐contrast MRI showed potential for the evaluation of CSF flow and spinal cord motion across a spinal stenosis [42, 43, 44]. However, a full understanding of CSF flow represents a challenge without information about CSF pressure. Therefore, preclinical and translational studies may investigate advanced neuroimaging methods alongside CSF pressure assessments. Furthermore, jugular vein compression could be used in conjunction with advanced neuroimaging, namely, the non‐invasive Queckenstedt's test, to study the response of CSF flow to increased pressure. This requires awareness and understanding of the history of Queckenstedt's test, its limitations and potential. Another area of interest is the intraoperative monitoring of CSF dynamics to quantify effects from surgical decompression [45, 46] and spinal cord perfusion [47]. Currently, most studies focus on CSF pressure or cardiac‐related CSF pulsations, whereas the potential of an intraoperative Queckenstedt's test was not explored. Lastly, in clinical trials in spinal cord injury that use the intrathecal route for drug administration, such as anti‐Nogo‐A antibodies [48], Queckenstedt's test and other CSF pressure metrics could help to confirm unrestricted CSF flow [49]. There is one recently published case series in patients with degenerative spinal stenosis in which Queckenstedt's test was performed when clinical and imaging findings were ambiguous or when interpretation of MRI was impeded by metal artifacts [50]. Further research is required to evaluate if Queckenstedt's test is a biomechanical marker that can complement the structural perspective as provided by imaging.

Residents should be aware of Queckenstedt's test for several reasons. Firstly, learning from history can inform the future. This concerns the potential combination of Queckenstedt's test with non‐invasive imaging and during intraoperative CSF monitoring. Secondly, Queckenstedt's test provides insights into the biomechanical features of CSF dynamics, a subsidiary topic in the era of non‐invasive spinal cord imaging. Greater awareness of CSF dynamics can facilitate a critical view on anatomical MRI, which contains information neither on CSF pressure nor on flow. Incorporating these factors into the pathophysiological framework of spinal cord compression may help clinicians to cope with seemingly paradoxical clinical and imaging findings. Lastly, the history of Queckenstedt's test teaches us that every medical procedure, no matter how established it appears, should be subject to critique and improvement.

In conclusion, Queckenstedt's test was the first specific clinical diagnostic test for spinal canal obstruction involving the evaluation of CSF dynamics and was routinely used until spinal CT and MRI were established. Neurologists, neurosurgeons, and scientists active in the field of spinal disorders should be familiar with the milestone achievements resulting from Queckenstedt's test and the physiological framework revealed by it, which is still valid today.

## AUTHOR CONTRIBUTIONS

**Armin Curt:** Conceptualization; investigation; funding acquisition; writing – original draft; writing – review and editing; visualization; validation; methodology; software; formal analysis; project administration; resources; supervision; data curation. **Carl Moritz Zipser:** Conceptualization; investigation; writing – original draft; methodology; funding acquisition; validation; visualization; writing – review and editing; project administration; formal analysis; software; data curation; supervision; resources.

## FUNDING INFORMATION

This work was supported by the Swiss Paraplegic Foundation (Foko_2019_01); the Balgrist Foundation; International Foundation for Research in Paraplegia (P190); Olga Mayenfisch Foundation; Swiss National Science Foundation (Project Nr. 320,030–231,396).

## CONFLICT OF INTEREST STATEMENT

The authors have nothing to disclose.

## Data Availability

The data that support the findings of this study are available from the corresponding author upon reasonable request.
